# Temozolomide and Reirradiation in Recurrent Grade II Brain Glioma

**DOI:** 10.14740/wjon838w

**Published:** 2014-06-25

**Authors:** Mohammed A Osman

**Affiliations:** General Organization for Teaching Hospitals, and Institutes, Cairo, Egypt. Email: mmoneam@hotmail.com

**Keywords:** Glioma, Temozolomide, Reirradiation, Survival, Quality

## Abstract

**Background:**

This phase II trial was designed to assess the response rate, survival benefits and toxicity profile of temozolomide, and brain reirradiation using conformal radiotherapy (CRT) for recurrent grade II brain glioma. Between February 2006 and June 2009, 18 patients with recurrent low grade glioma, and two with recurrent ependymoma were enrolled in the study. Patients had to show unequivocal evidence of tumor recurrence on gadolinium-enhanced magnetic resonance imaging after failing conventional radiotherapy for initial disease.

**Methods:**

Patients were treated by temozolomide at a dose of 200 mg/m^2^/day for chemonaive patients, and at a dose of 150 mg/m^2^/day for previously treated patients, for 4 - 5 cycles. Then, patients underwent reirradiation by CRT at a dose of 30 - 40 Gy by conventional fractionation.

**Results:**

All the 20 patients were treated with temozolomide and reirradiation. Two patients achieved complete remission, and six achieved partial remission, with an overall objective response rate of 40%. The mean overall survival (OS) was 16 months (range, 6 - 24 months). The median OS was 15.5 months. Additionally, treatment significantly improved quality of life. Treatment was tolerated well with mild grade 1, 2 hematological toxicities, and nausea/vomiting in 15% and 39% of cycles, respectively.

**Conclusions:**

Temozolomide and CRT had an anti-tumor activity in recurrent grade II brain glioma, and represented a good treatment hope for such patients.

## Introduction

Recurrent brain glioma is an incurable disease. The great majority of malignant gliomas (at least 70%) recur after initial treatment [1].

Grade II brain gliomas include low grade glioma (LGG), ependymoma, low grade oligodendroglioma and mixed oligoastrocytma [2].

There are some genetic abnormalities linked with recurrence including deletion of CDKN2A, deletion of P14ARF, amplification/overexpression of the CDK4 gene and EGFR amplification/overexpression or PGFR overexpression [2, 3].

There is no standard treatment for recurrent low grade brain glioma. The prognosis of recurrent brain glioma is poor with few breakthroughs in management over the past few decades. Patients without treatment usually survive for few months [4].

Temozolomide as an imidazotetrazine agent is effective in recurrent glioma. Myelotoxicity (primarily neutropenia and thrombocytopenia) is the major adverse effect of temozolomide observed in number of clinical trials [5, 6].

The brain tolerance for reirradiation depends on several factors including dose per fraction, total dose administered, overall treatment time, time interval between primary treatment and reirradiation, volume of brain irradiated, adjunctive therapies, and other factors [7, 8].

A wide variety of radiotherapy (RT) techniques have been used for reirradiation including 3D conformal RT (CRT), intensity-modulated radiotherapy (IMRT), brachytherapy, stereotactic radiosurgery (SRS), fractionated stereotactic radiotherapy (FSRT) and hypofractionated stereotactic radiotherapy (H-FSRT) [8].

This study commenced in Ain Shams University, Cairo, Egypt, in 2006 as a prospective, open-label study for patients with recurrent brain glioma.

### Study objectives

The primary end point of the current trial was response. The secondary end points were overall survival (OS), progression-free survival (PFS), the quality of life (QoL) and toxicity.

### Patients and Methods

Patients with histological proven LGG (grade 2) were eligible for the study if they had unequivocal evidence of tumor recurrence as shown by gadolinium-enhanced magnetic resonance imaging (MRI) after failing conventional RT, with or without chemotherapy for initial disease.

Additionally, inclusion criteria included Karnofsky performance status (KPS) ≥ 60, and aged above 18 and below 70 years. Patients were also required to have adequate hematological, renal and liver functions. All patients must have had received RT as part of their initial therapy. An interval of at least 3 months must have elapsed since the completion of the most recent course of radiation therapy, while at least 3 weeks must have elapsed since the completion of a non-nitrosourea containing chemotherapy regimen and at least 6 weeks since the completion of a nitrosourea containing chemotherapy regimen. Additionally, patients must have no concurrent malignancy. A written consent was taken from the patients prior to enrollment.

The study was conducted in the Oncology Unit, Ain Shams University Specialist Hospital (ASUSH) in the period between February 2006 and June 2009.

### Treatment protocol

**Figure 1:**
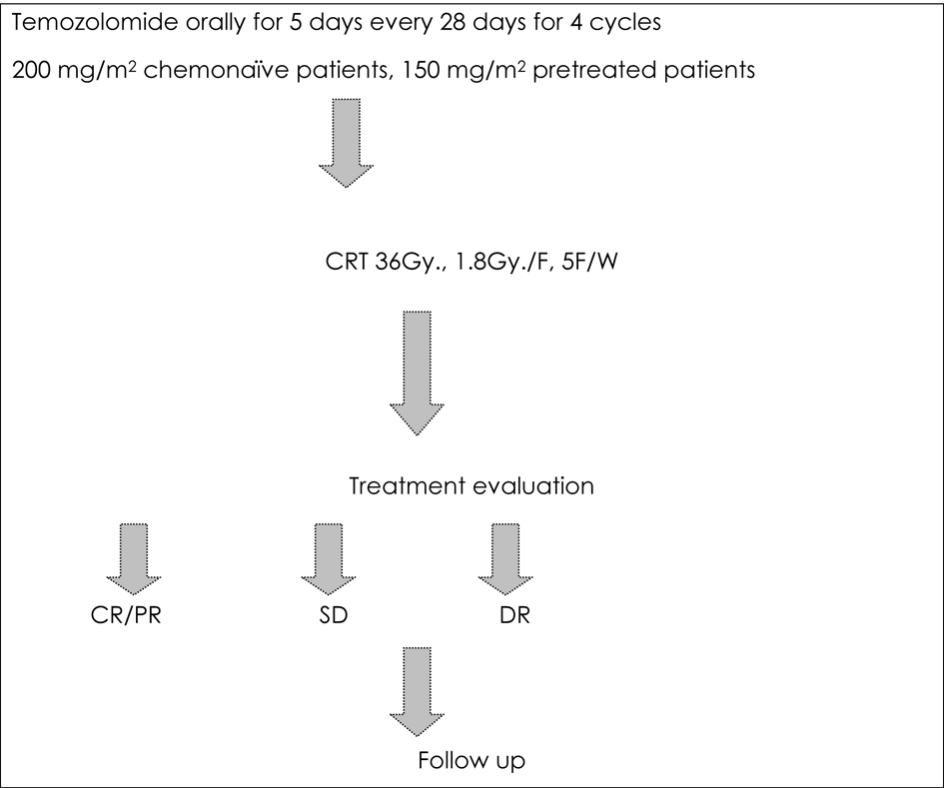
Treatment protocol for the current study.

The study design is shown in Figure 1. While on temozolomide, patients were advised to receive prophylaxis against pneumocystis carinii pneumonia (PCP) especially in the first two cycles, and antiemetic prophylaxis from day 1 to day 10 of each cycle.

#### Technique of reirradiation

Reirradiation was done using 3D CRT.

#### Patient positioning and immobilization

**Figure 2:**
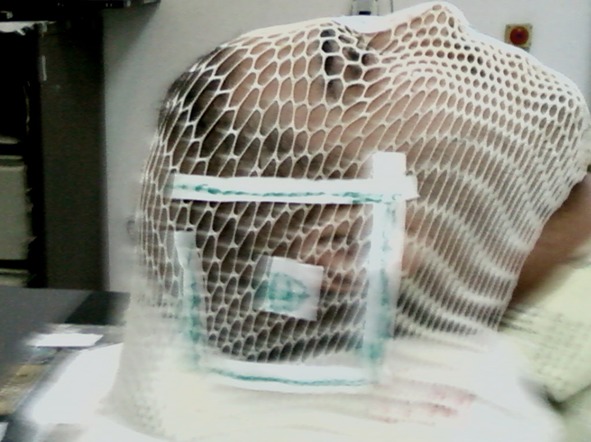
Patient simulation and immobilization.

Supine with neutral neck position. Immobilization was maintained with the use of the commercially available thermoplastic devices (Fig. 2).

#### Target localization

Target volume was defined by MRI data in corporation with treatment planning CT.

#### Volumes definition

T1 images on MRI were used to define the gross tumor volume (GTV). T2-weighted and FLAIR images were used to define the clinical target volume (CTV). The planning target volume (PTV) was defined by adding 1 cm to the GTV to include the surrounding edema. The PVT was reduced in areas near organ at risks (OARs). These OARs included the eyes, optic nerves, optic chiasm and brainstem. The beams directions, portals were defined for adequate and homogenous coverage of the target volume.

The target was considered to be appropriately treated if the PTV was enclosed within the 95% to 105% isodose line.

During RT, patients were kept on dexamethasone 4 mg orally, every 8 h, with PPI 40 mg orally, once daily, and antiepileptic prophylaxis.

#### Evaluation

Patients were evaluated after treatment protocol for both subjective and objective response (Table 1). MRI of brain (or CT if MRI was medically contraindicated) was done after chemotherapy cycles, after treatment protocol, and every 2 months in first 6 months, then every 3 months thereafter for follow-up.

**Table 1:** Response Definitions

Complete response (CR)	Disappearance of all contrast-enhancing tumor.
Partial response (PR)	50% or more reduction in the size of measurable disease.
Disease progression (DP)	25% or more increase in the size of measurable disease.
Stable disease (SD)	All other situations.

#### QoL evaluation

The trial applied FACT/NCCN (The Functional Assessment of Cancer Therapy/National Cancer comprehensive network) brain symptom index (FBrSI)-15 questionnaires to assess the QOL. The FBrSI-15 questionnaires (after translation to Arabic) were asked to patients in four occasions: at start of treatment (baseline), after the end of treatment protocol, 4 weeks after treatment and 4 weeks thereafter.

Improved QoL scores were defined as clinically significant if a change of the score of 4 points or more.

#### Toxicity profiles

Toxic effects were graded according to the National Cancer Institute Common Toxicity Criteria, version 2.0.

Repeat cycles of chemotherapy were administered on schedule only if the patient met the following re-treatment criteria (Table 2).

**Table 2:** Dose Modification Based on Hematological and Renal Function Results

Hematology			
For day 1			
Neutrophils (10^3^/mL)		Platelets (10^3^/mL)	Dose
≥ 1.5	and	> 100	100%
< 1.5	or	< 100	Delay
Renal function test			
Creatinine			Dose
< 2 upper normal limit			100%
≥ 2 upper normal limit			Delay
Liver function tests			
Bilirubin (µmol/L)		ALT, AST	Dose
≤ 25	and	≤ 2.5 upper normal limit	100%
> 25	or	> 2.5 upper normal limit	Delay

### Statistical analysis

All calculations were carried out using SPSS (Statistical Package for Social Sciences) software for windows, version 15 (SPSS Inc., Chicago, USA). The objective response rate (ORR) was calculated by pooling the CR, and PR rates. Mean and median values were used for the description of continuous data. OS and PFS were analyzed by the Kaplan-Meier method, with use of two-sided log-rank statistics. P value was significant at ≤ 0.05 levels, and insignificant at > 0.05.

## Results

Between February 2006 and June 2009, 20 patients with recurrent grade II brain glioma were recruited. All had radiological evidence of recurrence. The mean age was 29 years (range 18 - 50). The mean KPS was 87.5. There were 18 patients with recurrent LGG, and two with ependymoma. Of the 20 patients, 13 had temporal lobe lesions, six had parietal lesions and one had cerebellar disease. The mean tumor size was 2.5 cm, and the median was 2.5 cm (Table 3).

**Table 3:** Patients’ and Disease’s Characteristics of the Current Study

Characteristics	Details	Patients No.	Percentage (%)
Age	18 - 20	6	30
	> 20 - 40	11	55
	> 40 - 60	3	15
	> 60	0	0
Sex	Male	15	75
	Female	5	25
Performance status	100	3	6.7
	90	9	23.3
	80	8	43.3
	70	0	23.3
	60	0	3.3
Pathology	LGG	18	90
	Ependymoma	2	10
Site of recurrence	Temporal lobe	13	65
	Parietal lobe	6	30
	Cerebellar	1	5
Pretreatment tumor size	1 - 2 cm	9	45
	2 - 4 cm	11	55
	> 5 cm	0	0

**Figure 3:**
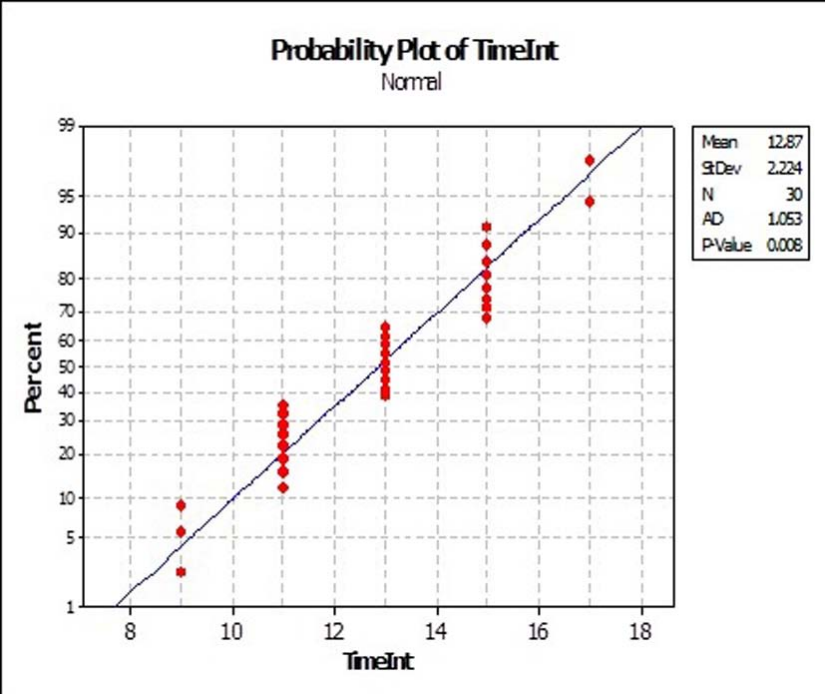
Time interval in the current series.

All the 20 patients had received RT treatment in the management of their primary lesion at a dose range of 55 - 70 Gy. Three patients received previous chemotherapy (one PCV and two temozolomide). The time interval between the two RT courses was in the range of 8 to 18 months, with a mean of 12.8 months, and a median of 13 months (Fig. 3).

All patients underwent the treatment protocol. The main cycles of temozolomide were 3.5 (range, 3 - 5 cycles). Patients were given reirradiation after the end of the last chemotherapy cycle by mean time of 2.5 weeks (range, 2 - 3 weeks). The main RT dose delivered was 36 Gy (range, 30 - 40 Gy).

### Response data

Objective responses (CR, PR) were observed in eight out of 20 patients. Two patients achieved CR (two LGG). Six patients achieved PR (five LGG and one ependymoma), with an overall ORR of 40%. SD was achieved in eight patients (seven LGG and one ependymoma), with an SD rate of the study 40%. DP was observed in the remaining four patients with DP rate of the study 20% (Table 4).

**Table 4:** Characters of the Eight Patients Who Achieved Objective Responses

Patient number	Response achieved	Age	Sex	PS	Pathology type	Time interval
1	CR	27	male	100	LGG	15
2	CR	20	male	90	LGG	17
3	PR	18	female	90	LGG	12
4	PR	18	male	100	LGG	15
5	PR	18	male	90	Ependymoma	13
6	PR	31	male	90	LGG	16
7	PR	19	female	100	LGG	17
8	PR	32	male	90	LGG	12

### Survival data

The mean OS was 16 months (range, 6 - 24 months). The median OS was 15.5 months. The mean PFS of the study group was 14.1 months (range, 5 - 24 months). The median PFS was 14 months.

**Figure 4:**
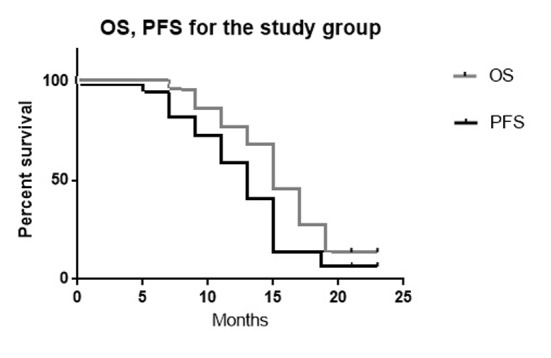
The Kaplan-Meier curves of OS and PFS for the current study (P value 0.04).

The 6-month OS was 100%. The 6-month PFS was 95%. The 12-month OS was 85%. The 12-month PFS was 75% (Fig. 4, Table 5).

**Table 5:** The 12-Month OS for Treatment Group Grouped by Prognostic Factors

12-month OS	Number (%)	P value
Age		
< 40 years old	16 (94%)	0.001
> 40 years old	1 (33%)	0.001
Performance status		
100-90	11 (91%)	0.6
80	6 (75%)	0.6
Tumor size		
< 2 cm	8 (89%)	0.03
> 2 cm	9 (81%)	0.03
Time interval between the two RT courses		
8 - 12 months	5 (71%)	0.001
12 - 16 months	11 (91%)	0.001
16 - 18 months	1 (100%)	0.001

### QoL data

After analysis of QoL using FBrSI-15 questionnaires, we observed before treatment (at baseline) that, after assessment of 19 patients who respond to the questionnaires, the mean score was 35/60 (range, 30/60 to 38/60). Just after treatment, the mean score was 24/60 (range, 18/60 to 30/60).

At 4 weeks after treatment (after assessment of the 16 patients who achieved ORR or SD), the mean score was 20/60 (range, 14/60 to 24/60). At 4 weeks thereafter (after assessment of 16 patients), the mean score was 21/60 (range, 14/60 to 24/60).

### Toxicity profile

Toxicities were evaluated in all the 20 patients enrolled in the study. Chemotherapy-related side effects included, among 76 chemotherapy cycles given, grade 1 or 2 toxicity hematological toxicity that occurred in 12 cycles (15%). Grade 1, 2 neutropenia was observed in seven cycles. Grade 1, 2 thrombocytopenia was observed in five cycles. No patient required dose reduction or cycle delay.

Other non-hematologic toxicities included grades 1, 2 toxicity in the form of nausea and vomiting that occurred in 30/76 of cycles (39%).

#### RT-related side effects

Among the 20 patients treated by CRT, no acute neurologic toxicity of grade II or higher was observed. Only, minor temporary acute radiation side effects included alopecia, headaches, grade 1, 2 nausea/vomiting responded to antiemetics, and skin erythema. There was no late radiation morbidity observed in the follow-up period.

## Discussion

Recurrent brain glioma is an incurable disease. Treatment options for recurrent brain glioma include chemotherapy, radiation therapy, surgery and palliation [9].

One of the main issues in treating recurrent brain glioma is the principal decision if a definitive therapy is appropriate or just palliative care. Another issue is the best treatment regimen. The main goal of surgery is removal of the enhancing tissues to decrease pressure effect and to provide diagnosis in ambiguous cases [9].

Temozolomide as an imidazotetrazine agent appears effective in patients with recurrent brain glioma. It is associated with ORR of 40% in LGG [5, 6, 10-11].

In the current study, 20 patients with recurrent low grade brain glioma were treated by combined modality using temozolomide for four cycles, then CRT. Most of the clinical researchers prefer to give temozolomide alone instead of chemoradiation in recurrent brain glioma that may be attributed to two important reasons. 1) Toxicity of combined modality is expected to be much higher than each modality. However, from literature reviews, when comparing the rate of severe toxicity (grade 3, 4) by temozolomide alone with that of temozolomide plus reirradiation, it was in the range of 6-18% and 11-19% respectively. 2) Fear of giving two irradiation treatment courses in short time interval (< 12 months), due to insufficient data that cover this point of interest [12-14].

When reviewing trials published within the last 6 years in recurrent brain glioma, it was found that in order to achieve a good objective response, and survival benefit, researchers used either temozolomide for median of eight cycles alone, or temozolomide for median of 4 - 6 cycles plus other agents including RT [14, 15].

It was avoided to give temozolomide concurrently with RT, in order to avoid unexpected toxicities in those previously irradiated patients.

In the current study, 20 Egyptian patients showed ORR, and survival benefit comparable with other studies done in Caucasian patients [11, 16, 17].

In the current study, the QoL data were analyzed as important information about the response to treatment. A subjective response is considered important factor for treatment response, and sometimes as important as the objective response in recurrent brain glioma. One of the aims of treatment of such cancers is to allow patients to enjoy their life without significant neurological deterioration, seizures, or psychological troubles. The current treatment protocol significantly improved the QoL in patients with recurrent brain glioma.

The current treatment protocol was well tolerated, with only mild side effects. Temozolomide had demonstrated a safe drug profile in the current study, with mild emetogenic effects during the days of drug ingestion (days 1-5), and was relieved by anti-emetics. The drug had reversible myelosuppression (neutropenia, and thrombocytoprnia). Nadir platelet and neutrophil counts typically occurred on days 21 to 26 of the cycles. No grade 3 or 4 toxicity was recorded.

Reirradiation had no acute neurologic toxicity of grade II or higher. Only observed, minor (grade 1) temporary acute radiation side effects included headaches, mild (grade 1, 2) nausea/vomiting responded to antiemetics, and skin erythema. There was no late radiation morbidity observed.
